# Ewing’s Sarcoma of the Calcaneus

**DOI:** 10.7759/cureus.95412

**Published:** 2025-10-25

**Authors:** Hafsa Jamal Eddine, Othmane Zouiten, Leila Afani, Mohamed El Fadli, Rhizlane Belbaraka

**Affiliations:** 1 Medical Oncology, Mohammed VI University Hospital, Marrakech, MAR

**Keywords:** calcaneus, ewing’s sarcoma, malignant tumor, metastasis, round cell tumor

## Abstract

Ewing’s sarcoma (ES) is a rare malignant bone tumor that usually affects long bones and the pelvis and is most frequently diagnosed during childhood and adolescence.

We report the case of a 30-year-old woman who presented with calcaneus pain and swelling. At the time of diagnosis, imaging tests showed a calcaneal lesion with bone metastases. ES was confirmed by histopathological examination. The patient was treated with palliative chemotherapy and surgery due to the progression of the swelling and local secondary infection. Postoperative computed tomography of the thorax, abdomen, and pelvis revealed lung and bone progression. Second-line chemotherapy was planned, but unfortunately, the patient died of pulmonary embolism.

This case illustrates the aggressive nature of calcaneal ES and emphasizes the importance of early recognition and comprehensive evaluation.

## Introduction

Ewing’s sarcoma (ES) is the second most common primary bone malignancy following osteosarcoma, typically affecting adolescents and young adults. They have diaphyseal or metadiaphyseal origin, including the most common primary sites of long bones (47%), pelvis (26%), chest wall (16%), and spine (6%). Involvement of the small bones of the foot is extremely rare [1]. There have also been documented cases that involve the talus, demonstrating the unusual location of this tumor in the foot [2]. Calcaneal involvement is even more exceptional, with only a few cases documented in the literature. We give a thorough analysis of the pertinent literature and describe the case of a 30-year-old woman who was diagnosed with ES of the right calcaneus. 

## Case presentation

This case concerns a 30-year-old woman who presented to the orthopedic center complaining of right heel pain and swelling that had persisted for six months, accompanied by an unintentional 6 kg weight loss. Clinical examination revealed a tender bony mass in the right foot, painful and restricted movement, without any distal neurovascular deficit. Vital signs were within normal limits. There was no significant medical history and no occupational exposures. The results of the laboratory tests fell within the norm (Table 1).

**Table 1:** Summary of key laboratory findings with corresponding reference ranges and interpretation

Parameter	Patient value	Reference range	Interpretation
Hemoglobin ( g/dL)	12	12-16	Normal
White blood cell count (/mm³)	9,700	4,000-11,000	Normal
Platelet count (/mm³)	344,000	150,000-400,000	Normal
C-reactive protein (mg/L)	4.8	<5	Normal
Urea (g/L)	0.15	0.15-0.4	Normal
Creatinine (mg/L)	5	5-9	Normal
Aspartate aminotransferase (IU/L)	18	<40	Normal
Alanine aminotransferase (IU/L)	19	<40	Normal
Total bilirubin (mg/L)	7	<10	Normal
Alkaline phosphatase (IU/L)	94	30-100	Normal

**Figure 1:**
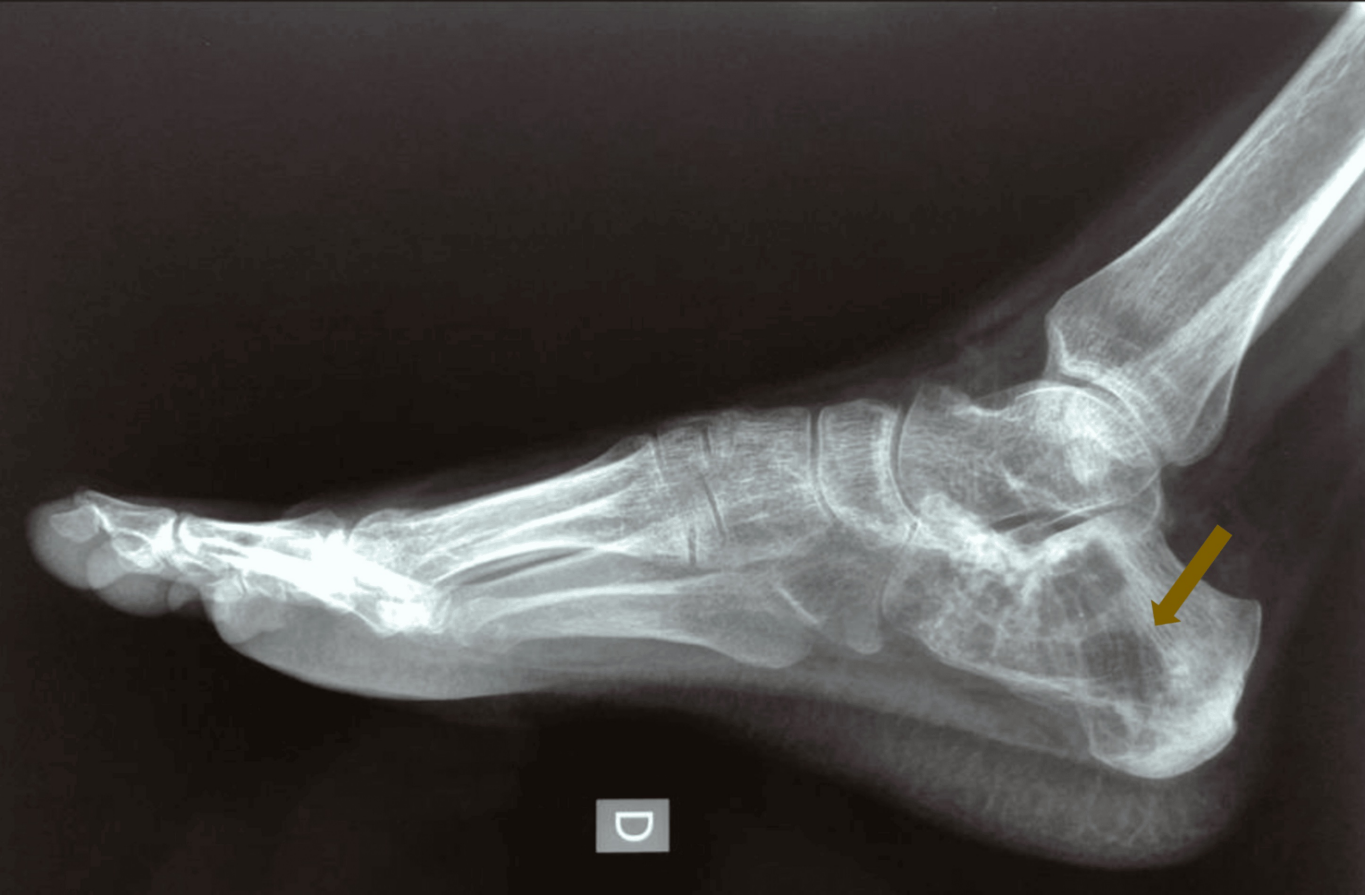
Plain radiograph of the right heel showing a well defined, expansile lytic lesion in the calcaneus. Well-defined, expansile lytic lesion in the calcaneus consistent with aggressive bone tumor; absence of periosteal reaction suggests a primary malignant lesion rather than infection.

Plain radiography of the right heel identified an extensive lytic lesion in the calcaneus without evidence of cortical disruption or periosteal reaction (Figure 1).

**Figure 2:**
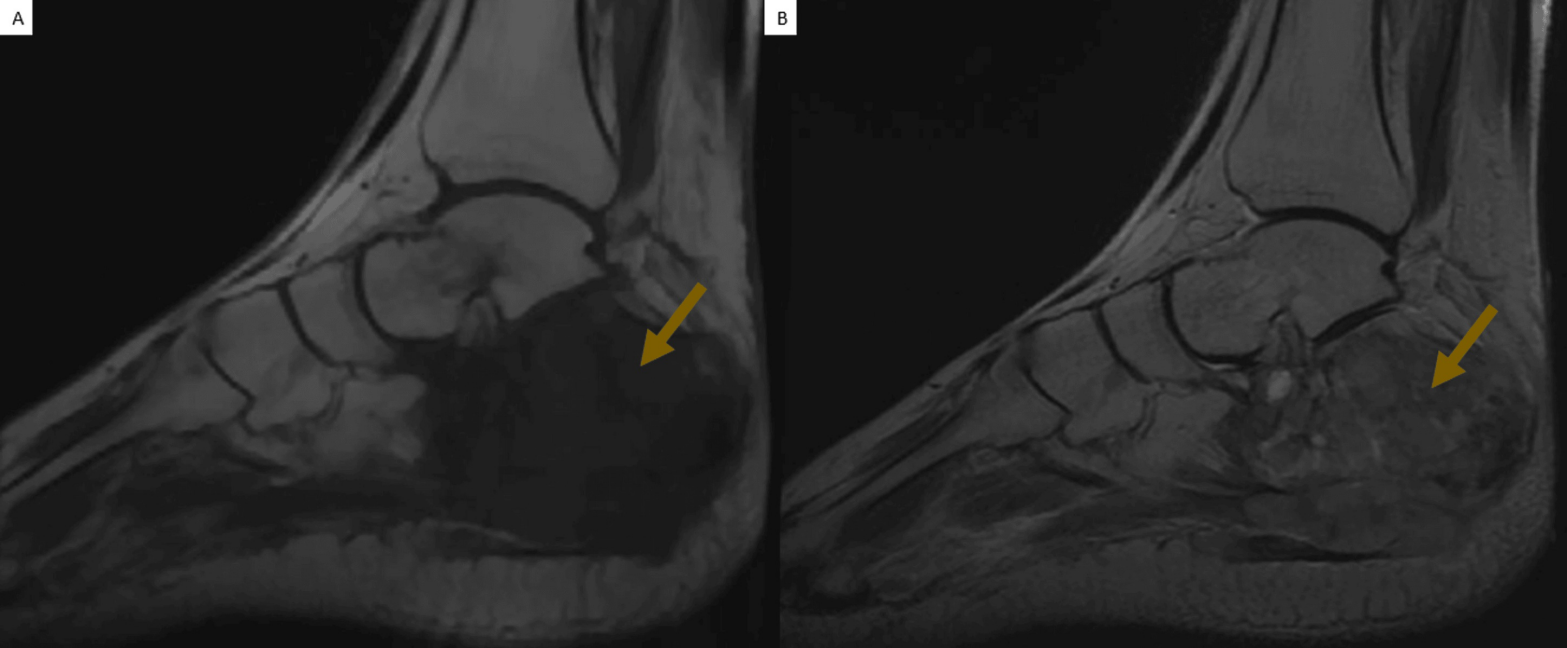
On T1-weighted sagittal MRI of the ankle (A), the lesion appeared hypointense. On T2-weighted imaging (B), the lesion was heterogeneously hyperintense. Significant post-contrast enhancement was also observed in these areas.

The right ankle's MRI demonstrated a calcaneal-dependent bone lesion process with irregular margins and focal cortical disruption. The lesion appeared hypointense on T1-weighted images and heterogeneous hyperintense on T2 sequences, heterogeneously enhanced after gadolinium contrast administration, delineating areas of central necrosis and local infiltration, which corresponded to aggressive histology and rapid clinical progression (Figure 2).

**Figure 3:**
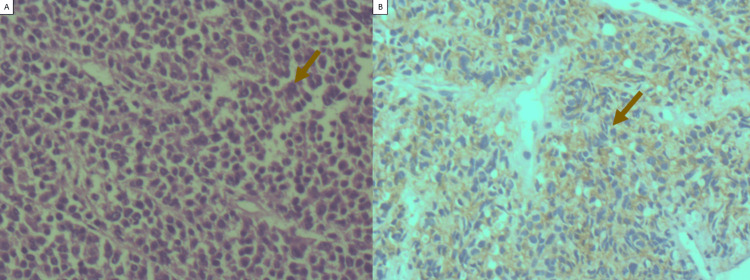
(A) Photomicrograph of the tumor stained with H&E (100×) showing sheets of small round cells with a high nucleus-to-cytoplasm ratio. (B) Immunohistochemistry revealing diffuse membranous positivity for CD99 in the tumor cells. CD99: Cluster of differentiation 99; H&E: Hematoxylin and eosin.

An ultrasound-guided biopsy was performed. The lesion's histological examination revealed a malignant tumor made up of uniformly distributed, small, round cells. Significant membranous positivity for CD99 was identified by immunohistochemical analysis (Figure 3).

**Figure 4:**
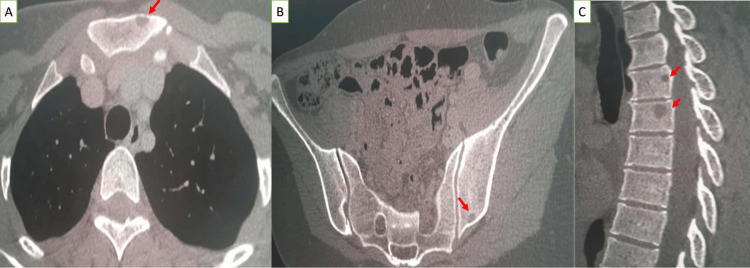
On the bone window of the CT scan, multiple osteolytic lesions without marginal sclerosis were classified as Lodwick Ib visible in the manubium of the sternum (A), the right iliac (B), and the vertebral bodies of the dorsolumbar spine (C).

A computed tomography of the thorax, abdomen, and pelvis (CT-TAP) revealed bone metastases, leading to the diagnosis of metastatic ES of the calcaneus (Figure 4).

The patient received four cycles of alternating chemotherapy regimens: vincristine (1.4 mg/m²), doxorubicin (75 mg/m²), and cyclophosphamide (1.2 g/m²) (VDC), alternating with ifosfamide (9 g/m²) and etoposide (500 mg/m²) (IE). The patient showed no clinical improvement, with progression of swelling and local secondary infection. She then underwent below‑knee amputation. Postoperative CT-TAP revealed lung and bone progression. Second-line chemotherapy with etoposide and cisplatin was planned, but unfortunately, the patient died of pulmonary embolism.

## Discussion

The second most common primary bone cancer is ES, which usually affects children and teenagers aged 5 to 20 years. It usually starts in the diaphysis of long bones and the axial skeleton. Rarely do small foot bones get involved [3]. Calcaneus involvement is observed very rarely, with few cases reported in the literature. A summary of these reports, along with the present case, is presented in Table 2.

**Table 2:** Summary of case reports on Ewing's sarcoma of the calcaneus BKA: Below-knee amputation; CT: Chemotherapy; F: Female; M: Male; NR: Not reported; RT: Radiation therapy.

Author/year	Age/sex	Symptoms	Duration (months)	Metastasis	Treatment	Survival (months)
Choi et al. (2004) [4]	15 yr/F	Pain and swelling	8	0	CT and BKA	22 (on follow-up)
Madhar et al. (2008) [3]	14 yr/M	Pain and swelling	12	0	CT and BKA	6
Zarei et al. (2016) [5]	13 yr/F	Pain and swelling	8	Lung	CT, RT, and BKA	24 (on follow-up)
Guarnieri et al. (2016) [6]	23 yr/F	Pain and swelling	12	Bone and lung	CT	NR (deceased)
Sherif et al. (2017) [7]	10 yr/F	Pain and swelling	2	0	CT and BKA	NR (on follow-up)
Sahin et al. (2018) [8]	19 yr/F	Pain, swelling, and mass	6	Inguinal and popliteal lymph nodes	CT and BKA with lymph node dissection	12 (on follow-up)
Torner et al. (2022) [9]	13 yr/M	Pain and swelling	6	0	CT and calcaneal reconstruction with total calcaneus allograft	42 (on follow-up)
Mohanty et al. (2022) [1]	12 yr/F	Pain and swelling	4	0	CT and BKA	NR (on follow-up)
Tos et al. (2023) [10]	12 yr/M	Pain and swelling	18	0	CT and resection	NR (on follow-up)
Present case	30 yr/F	Pain and swelling	6	Bone and lung	CT and BKA	9

The mean age of reported cases, according to a review of the literature, was 16.1 years, with a range of 10 to 30 years. There was a female predominance with a sex ratio of 2.33. Ankle pain and swelling were the most common symptoms, and they endured for a mean of 8.2 months.

Histologically, ES consists of sheets of small round cells with a high nucleus-to-cytoplasm ratio. The cells have round nuclei with finely distributed chromatin, with one or more tiny nucleoli and little eosinophilic cytoplasm [1]. Its differential diagnosis includes other small round cell malignancies, particularly small cell osteosarcoma, mesenchymal chondrosarcoma, lymphoma, and metastatic neuroblastoma [4]. To distinguish ES from other small round cell tumors, immunohistochemistry is crucial. Membranous CD99 expression and nuclear FLI1 staining are common features of classic ES cells. Neuron-specific enolase, synaptophysin, and S100 protein may also be expressed by tumor cells, depending on the degree of neuroectodermal differentiation. Through the use of fluorescence in situ hybridization to identify ES/PNET gene rearrangements and the distinctive reciprocal translocation t(11;22), molecular testing can validate the diagnosis [1].

The radiologic imagery showed that 40% of the patients had distant metastases when they first arrived. The majority of reported cases received chemotherapy. Seven patients had below-knee amputations, and one patient had calcaneal reconstruction with a total calcaneus allograft.

The use of adjuvant or neoadjuvant chemotherapy was used in eight cases. The palliative chemotherapy was used in two cases. VDC cycles alternate with IE in the standard chemotherapy regimen [11].

Disease stage, tumor location and size, response to chemotherapy, and treatment modality are important prognostic factors for ES. Compared to lesions in other areas of the hands or feet, calcaneus tumors seem to have a worse prognosis [4]. Patients with metastatic ES at presentation fare much worse, with five-year survival rates of about 80% for localized disease and 19%-30% for those with bone metastases [12].

## Conclusions

ES of the calcaneus is extremely rare. Diagnosis is often delayed due to its unusual location and non-specific clinical presentation, which may negatively impact prognosis, especially in cases with metastatic spread. ES can be distinguished from other tumor types through histopathological confirmation, which remains essential for diagnosis. Treatment is guided by a multidisciplinary approach. For localized disease, chemotherapy and radical surgery are generally recommended, while palliative chemotherapy is the main option for metastatic or locally advanced stages, based on literature and guidelines. In general, the prognosis of this disease remains guarded.
